# Sub-milliwatt threshold power and tunable-bias all-optical nonlinear activation function using vanadium dioxide for wavelength-division multiplexing photonic neural networks

**DOI:** 10.1038/s41598-025-90350-3

**Published:** 2025-02-15

**Authors:** Jorge Parra, Juan Navarro-Arenas, Pablo Sanchis

**Affiliations:** 1 Nanophotonics Technology Center, Universitat Politècnica de València, Camino de Vera s/n, 46022 Valencia, Spain; 2 Institute of Materials Science (ICMUV), Universitat de València, Carrer del Catedràtic José Beltrán Martinez 2, 46980 Valencia, Spain

**Keywords:** Integrated optics, Silicon photonics, Photonic devices, Materials for devices

## Abstract

The increasing demand for efficient hardware in neural computation highlights the limitations of electronic-based systems in terms of speed, energy efficiency, and scalability. Wavelength-division multiplexing (WDM) photonic neural networks offer a high-bandwidth, low-latency alternative but require effective photonic activation functions. Here, we propose a power-efficient and tunable-bias all-optical nonlinear activation function using vanadium dioxide (VO_2_) for WDM photonic neural networks. We engineered a SiN/BTO waveguide with a VO_2_ patch to exploit the phase-change material’s reversible insulator-to-metal transition (IMT) for nonlinear activation. We conducted numerical simulations to optimize the waveguide geometry and VO_2_ parameters, minimizing propagation and coupling losses while achieving a strong nonlinear response and low-threshold activation power. Our proposed device features a sub-milliwatt threshold power, a footprint of 5 μm, and an ELU-like activation function. Moreover, the bias of our device could be thermally tuned, improving the speed and power efficiency. On the other hand, performance evaluations using the CIFAR-10 dataset confirmed the device’s potential for convolutional neural networks (CNN). Our results show that a hybrid VO_2_/SiN/BTO platform could play a prominent role in the path toward the development of high-performance photonic neural networks.

**Supplementary Information:**

The online version contains supplementary material available at 10.1038/s41598-025-90350-3.

## Introduction

Artificial neural networks (ANNs), inspired by biological brains, have revolutionized computational capabilities^1^, showcasing remarkable performance across a wide range of applications such as speech recognition^2^, image classification^3^, computer vision^4^, and natural language processing^5^. Hence, ANNs have achieved human-level accuracy in tasks that are challenging for traditional computers but relatively easy for humans. In this context, the rapid advancement of ANNs has driven the need for more efficient hardware to handle their increasing complexity and computational demands^6,7^. Traditional electronic-based systems, while having powered many of these advancements, face inherent limitations in speed and energy efficiency due to the nature of electronic devices, which suffer from limited bandwidth and susceptibility to interference. As Moore’s Law approaches its limits, with transistor densities nearing their physical boundaries and Dennard scaling no longer providing the expected energy efficiency gains^8^, the performance growth of electronic processors has stagnated. This has led to a bottleneck in high-performance computing, where the demand for processing power continues to grow exponentially^9^, particularly driven by the proliferation of artificial intelligence (AI)^10^ and machine learning applications^11^.

Photonic technologies present a promising solution to these limitations by leveraging light benefits^12,13^. Light offers matchless advantages in terms of bandwidth, latency, and energy efficiency. In telecommunications and data centers, optical interconnects have already established themselves as superior communication mediums due to their high-capacity and low-loss characteristics^14–16^. Therefore, this potential could be extended to information processing and computing, particularly in the domain of ANNs^17^.

Integrated photonics are well-suited for high-performance implementations of ANNs due to their advantages in interconnectivity and linear operations^18,19^. Connections between pairs of artificial neurons can be represented as matrix-vector operations, with optical signals being multiplied through tunable waveguide elements and summed using wavelength-division multiplexing (WDM)^20–23^. Silicon photonics, in particular, offers a cost-effective and scalable platform, benefiting from the existing infrastructure of complementary metal oxide semiconductor (CMOS) fabrication^24–26^. However, integrated photonic neural networks still face some challenges, such as weak optical nonlinearity and the lack of suitable configurations for photonic hardware.

Strong optical nonlinearities are desired to build photonic hardware providing nonlinear activation functionalities into the neural network, thereby enabling them to model complex relationships. Current waveguide-based photonic solutions for activation functions face challenges and trade-offs related to device footprint, optical bandwidth, power threshold for activation, and dynamic tunability^27–36^. Hence, these limitations hinder the full realization of high-performance, scalable photonic neural networks. A promising approach to address these challenges could be the utilization of vanadium dioxide (VO_2_), a phase-change material known for its reversible insulator-to-metal transition (IMT) when stimulated optically^37,38^. The IMT in VO_2_, characterized by substantial changes in electrical conductivity and optical transmittance near room temperature (~ 65 °C), presents a unique mechanism for achieving all-optical photonic devices with ultra-compact footprint and broad spectral operation such as switches^39–41^, optical limiters^42^, or photonic memories^43–45^. On the other hand, new CMOS-compatible integrated photonic platforms have emerged in recent years, such as the hybrid silicon or silicon nitride (SiN) – barium titanate (BTO) platform^46,47^. The SiN-BTO platform may offer significant advantages for neuromorphic computing by combining the unique properties of BTO and SiN to enable high-performance photonic integrated circuits with high operation speeds and low transmission losses^48^. In this manner, information could be transferred between electrons and photonics with ultralow heat dissipation using SiN/BTO modulators, whereas photonic information processing could be done in such VO_2_-based devices^49^.

In this work, we propose an all-optical nonlinear activation function device using VO_2_ integrated into a SiN/BTO waveguide for WDM photonic neural networks. Our approach leverages the photoinduced IMT property of VO_2_ to design a power-efficient and tunable bias activation function. Through numerical simulations, we optimized the waveguide geometry and VO_2_ parameters to minimize propagation and coupling losses while achieving a strong nonlinear response and low-threshold activation power. Additionally, performance evaluations using the CIFAR-10 dataset confirm the device’s potential for advanced photonic neural network applications.

## Results

### Working principle and types of VO_2_-enabled nonlinear activation functions

**Fig. 1:**
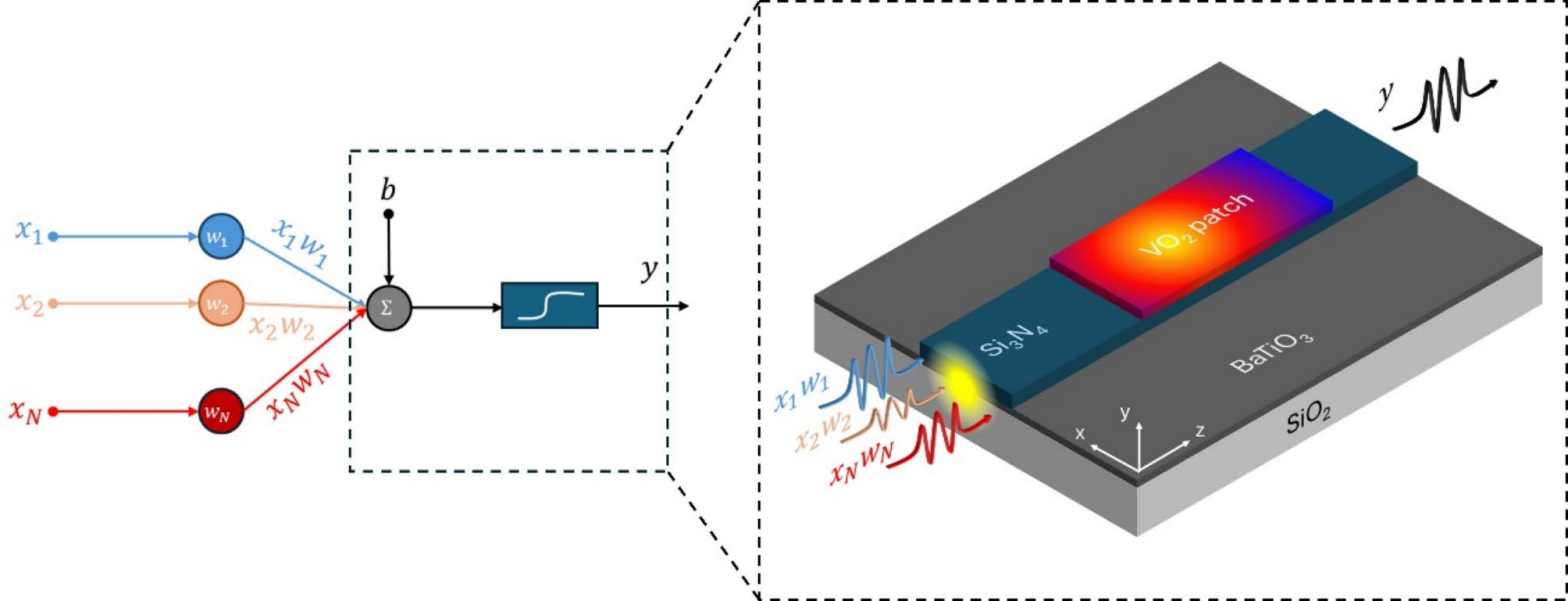
Sketch and working principle of the proposed activation function device enabled by VO_2_. The inputs, $\:{x}_{i}$, are encoded in different wavelengths and then weighted, $\:{w}_{i}$, accordingly by using a dot-product device before entering our device. The resulting weighted inputs are added, biased, and then evaluated by the nonlinear activation function. In our proposed device, the summation is produced by the absorption of the VO_2_, and thus photothermally triggering its IMT, which features a nonlinear change in the refractive index of VO_2_ with non-uniform heating in the VO_2_ patch as a function of the optical power. Therefore, the amplitude of the output signal, *y*, will depend on the inputs in a nonlinear way. Finally, the bias of the activation function is based on the background temperature of the VO_2_, which could be precisely controlled by using a microheater.

The working principle and the proposed activation function device are shown in Fig. 1. It consists of a SiN/BTO waveguide with a small VO_2_ patch on top. For this work, we consider a 1.1 μm × 150 nm SiN strip resting on an 80-nm-thick BTO layer^48^. The hybrid waveguide is covered with 1 μm-thick SiO_2_ cladding. Our proposed device works for transverse electric (TE) polarization at 1550 nm wavelength. A nonlinear response gives rise in the hybrid waveguide due to the photothermally-triggered IMT of VO_2_ caused by evanescent coupling of the different weights applied to the input WDM signal. Hence, a fan-in operation is enabled by the non-resonant geometry of the device, together with the broad spectral response of the VO_2_ refractive index at telecom wavelengths. On the other hand, the nonlinear input-output relationship can be engineered by tailoring the optical loss in the insulating and metallic state. The optical loss depends on the interaction between the optical mode and the VO_2_ patch; thereby, this value can be optimized by properly designing the geometry of the hybrid waveguide and fine-tuning its parameters, such as the VO_2_ width and thickness and the gap between the VO_2_ patch and the SiN/BTO waveguide.

For a VO_2_-based waveguide, the relationship between the input and output power, can be modeled as:

1 $$\:\begin{array}{c}{P}_{out}={P}_{in}-{\int\:}_{0}^{L}\alpha\:\cdot\:dz-2CL\end{array}$$

Where *P*_*out*_ is the output power in dBm, *P*_*in*_ is the input power in dBm, *α* is the propagation loss of the hybrid waveguide in dB/µm, *L* is the length of the hybrid waveguide in µm, and *CL* is the coupling loss between the SiN/BTO and the VO_2_/SiN/BTO waveguide in dB. The parameter *α* depends on both the optical power absorbed by the VO_2_, *P*_*abs*_, which is proportional to *P*_*in*,_ and the position of the VO_2_ patch along the propagation direction (z-axis), i.e., $\:\alpha\:=\alpha\:\left({P}_{abs},z\right)$, yielding a nonlinear input-output power relationship.

For the purpose of illustrating the potential to achieve various types of nonlinear activation functions enabled by VO_2_, we approximate the propagation loss variation between insulating to metallic to a step-function response by considering the abrupt IMT of VO_2_, thus:

2 $$L_{m} \approx \left\{ {\begin{array}{*{20}l} {0,} & {P_{{abs}} < P_{{IMT}} } \\ {\eta \left( {P_{{abs}} - P_{{IMT}} } \right),} & {P_{{abs}} \ge P_{{IMT}} } \\ \end{array} } \right.$$

where *α*_*i*_ and *α*_*m*_ are the propagation loss of the optical mode in the insulating and metallic state, respectively, and *P*_*IMT*_ is the optical power for which the VO_2_ undergoes the insulator-metal change. Moreover, by considering the exponential decay of the optical power along the propagation direction, the length of VO_2_ patch in the metallic state can be approximated as:

3 $$L_{m} \approx \left\{ {\begin{array}{*{20}l} {0, } & { P_{{abs}} < P_{{IMT}} } \\ {\eta \left( {P_{{abs}} - P_{{IMT}} } \right),} & {P_{{abs}} \ge P_{{IMT}} } \\ \end{array} } \right.$$

where $\:\eta\:$ is the thermo-optical coefficient, in µm/dB, relative to the rate conversion of the VO_2_ length from insulator to metal^42^. Therefore, by considering negligible coupling losses, Eq. (1) simplifies as:

4 $$P_{{out}} \approx \left\{ {\begin{array}{*{20}l} {P_{{in}} - \alpha _{i} L,} & {P_{{abs}} < P_{{IMT}} } \\ {P_{{in}} - \alpha _{i} L - \eta \varDelta \alpha {\varDelta}P,} & {P_{{abs}} \ge \:P_{{IMT}} } \\ \end{array} } \right.$$

$$\:\text{w}\text{h}\text{e}\text{r}\text{e}\:{\Delta\:}\alpha\:={\alpha\:}_{m}-{\alpha\:}_{i}\:\text{a}\text{n}\text{d}\:{\Delta\:}P={P}_{abs}-{P}_{IMT}.$$

**Fig. 2:**
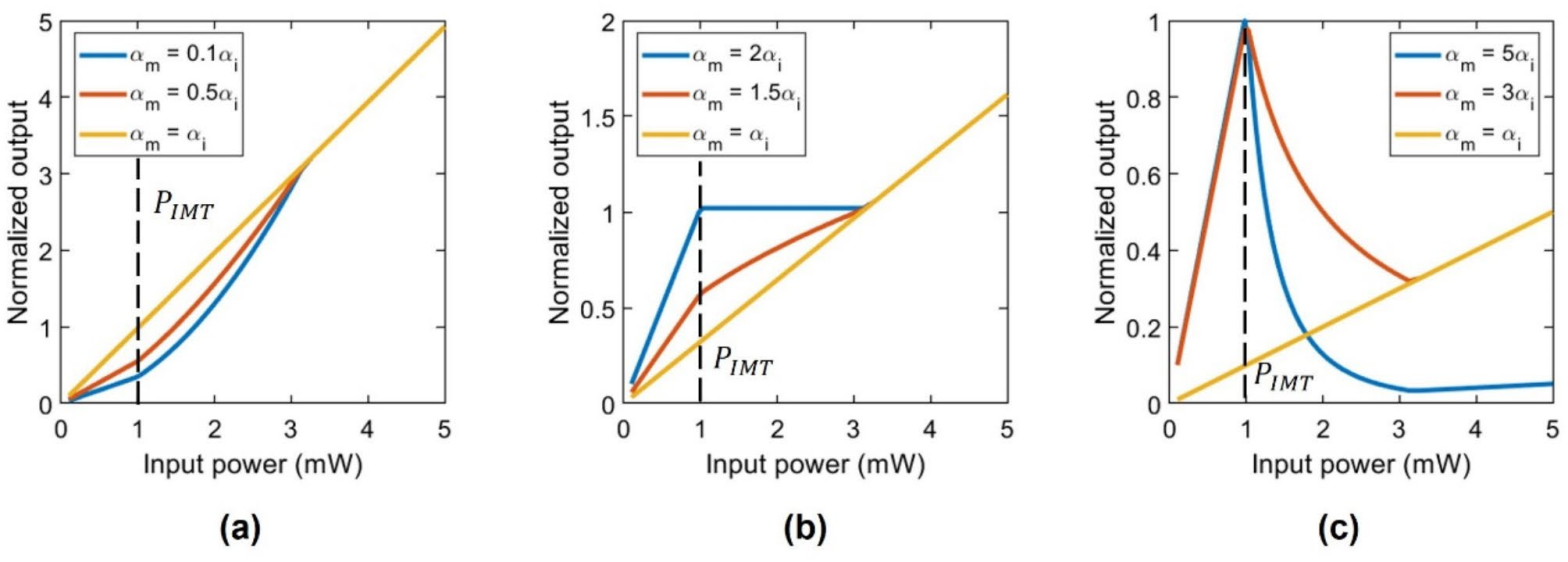
Nonlinear activation functions based on a waveguide device and enabled by VO_2_. (**a**) ELU shape for ηΔα < 0. (**b**) Clipped ReLu shape for ηΔα = 1 (α_m_ = 2α_i_). (**c**) Radial basis shape for ηΔα > 1. $\:{P}_{IMT}$ stands for the threshold power of the insulator-metal transition.

The value of $\:\eta\:{\Delta\:}\alpha\:$ sets the shape of the input-output relationship. Thus, different types of activation functions can be customized by properly designing the geometry of the hybrid waveguide and operation parameters such as polarization. Figure 2 illustrates the different types of nonlinear responses that can be achieved depending on the relation between the propagation losses in the insulating and metallic states. To obtain such responses, we employed Eq. (4), and considered the following values: $\:\eta\:=1$ µm/dB, $\:{\alpha\:}_{i}=1$ dB/µm, $\:{P}_{IMT}=1$ mW, and $\:L=5$ µm. It has to be noted, that due to the approximation of the IMT of VO_2_ to a digital step function Eq. (2), the transmission responses show abrupt and non-smooth variations in the transition points. The actual transition in a real VO_2_ device is very steep but analog. As a result, the abruptness of the transition is preserved, but the transmission response exhibits smoother and continuously differentiable. Therefore, despite the digital approximation, our model effectively captures the expected performance of a real device.

Therefore, for values of $\:\eta\:{\Delta\:}\alpha\:<0$ ($\:{\alpha\:}_{m}<{\alpha\:}_{i}$) an ELU-like activation function is obtained (Fig. 2a), where the output increases linearly and quasi-exponentially for $\:{P}_{in}<{P}_{IMT}$ and $\:{P}_{in}\ge\:{P}_{IMT}$, respectively, while it becomes linear for higher optical powers since the VO_2_ is fully metallic. If $\:\eta\:{\Delta\:}\alpha\:=1$ ($\:{\alpha\:}_{m}=2{\alpha\:}_{i}$) a flat response is achieved when the VO_2_ patch undergoes its IMT (Fig. 2b), thus resembling a clipped rectified linear unit (ReLU) activation function. On the other hand, if $\:\eta\:{\Delta\:}\alpha\:>1$ ($\:{\alpha\:}_{m}>{\alpha\:}_{i}$) a radial basis activation function is obtained (Fig. 2c) stemming from the linear response below the threshold power and the dramatic increase of the optical loss in the metallic state.

### Design of the optimal VO_2_/SiN/BTO hybrid waveguide for ELU activation function

**Fig. 3:**
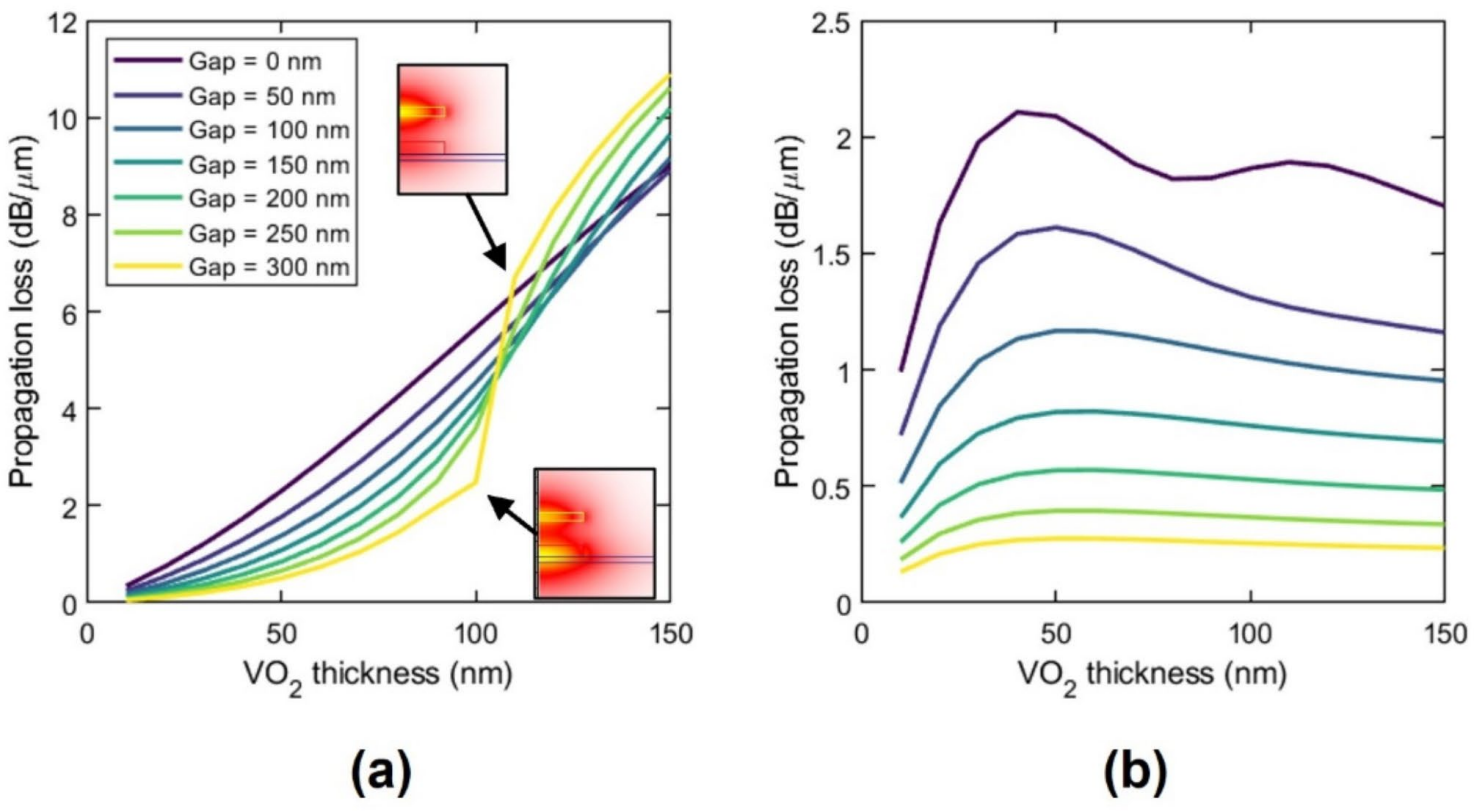
Propagation losses as a function of the VO_2_ thickness and gap for the (**a**) insulating and (**b**) metallic state.

In this work, we target the ELU-like response to design our activation function device. In order to achieve an ELU activation function featuring a strong nonlinear response and low-threshold activation power, the cross-section of the hybrid waveguide should be optimized to achieve large propagation loss in the insulating state while minimal loss in the metallic state, along with low coupling losses in both states. To this end, we calculated the propagation losses in the insulating and metallic states as a function of the VO_2_ thickness and gap (Fig. 3). We considered the VO_2_ patch, and the SiN strip had the same width. Propagation losses were calculated from the complex effective refractive index, *n*_*eff*_, of the fundamental optical mode supported by the hybrid waveguide (Supplementary note 1).

In the insulating state (Fig. 3a), as the VO_2_ becomes thicker, its interaction with the light increases since this is attracted toward the VO_2_ due to its higher real refractive index (~ 2.8) compared to SiN (~ 2) and BTO (~ 2.3). Eventually, the light is highly confined inside the VO_2_, yielding a significant increase in the propagation losses [see insets of Fig. 3a]. By contrast, in the metallic state (Fig. 3b), propagation losses show lower values and reach a plateau for thickness higher than ~ 50 nm, attributed to the small penetration depth of metallic VO_2_$\:{\delta\:}_{p}\approx\:\lambda\:/\left(4\pi\:{\kappa\:}_{m}\right)\approx\:47.5$ nm. Interestingly, for small gaps, the optical mode is pushed down toward the BTO layer as the VO_2_ thickens, resulting in a slight reduction of the propagation losses. Therefore, thick VO_2_ layers would be desired for fulfilling the propagation loss condition ($\:{\alpha\:}_{i}>{\alpha\:}_{m}$) of the ELU activation function. However, large optical mismatch between the SiN/BTO and VO_2_/SiN/BTO waveguides, and thus large coupling losses, are expected to arise for small gaps in the metallic state and for large gaps in the insulating state, thereby imposing a trade-off between low coupling losses and optimal propagation losses.

**Fig. 4:**
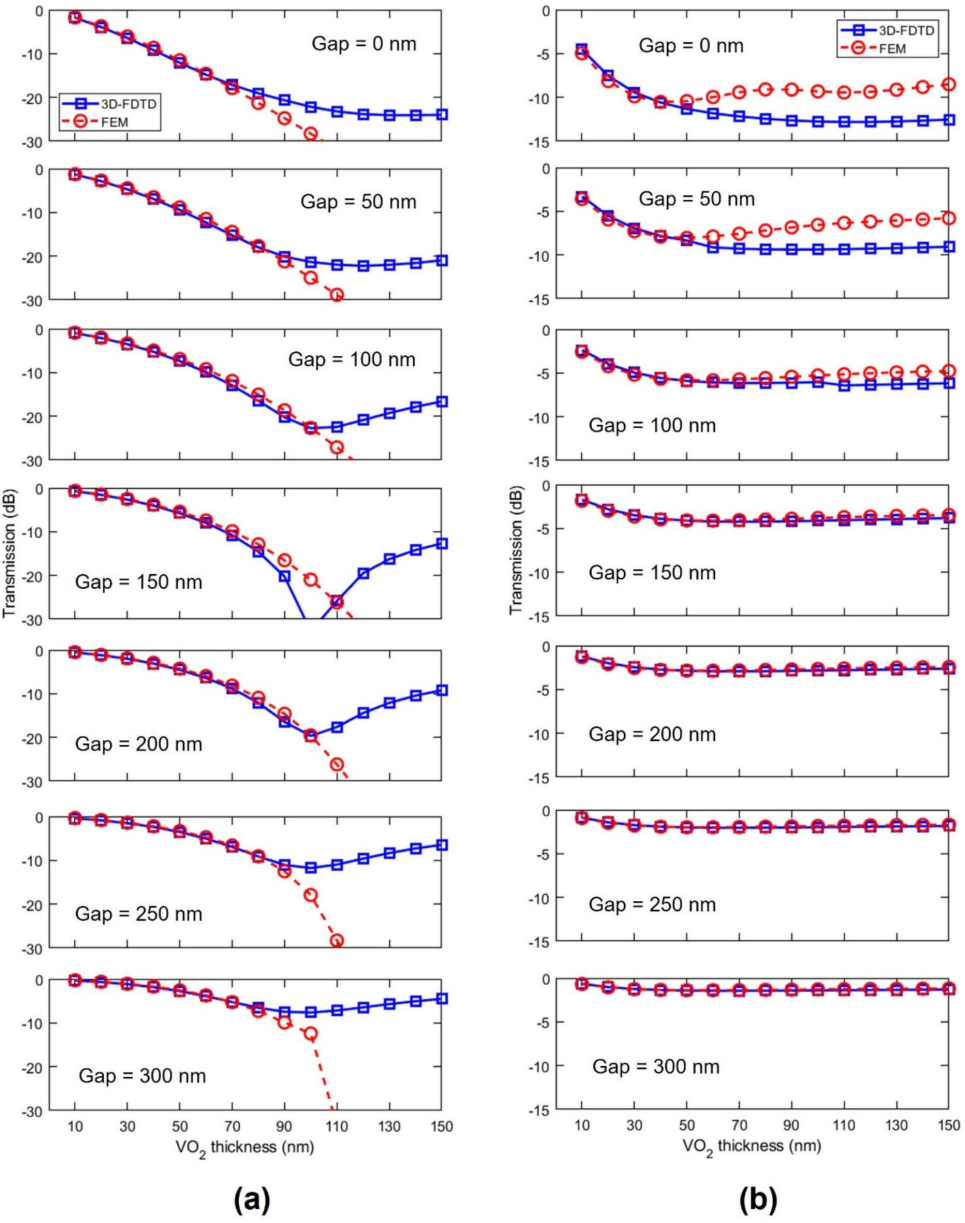
Transmission of 5-µm-long VO_2_/SiN/BTO waveguide as a function of the VO_2_ thickness and gap obtained by 3D-FDTD, and comparison with calculations considering only propagation losses obtained by FEM simulations. (**a**) Insulating and (**b**) metallic states.

In order to evaluate the impact of the optical mismatch, we calculated the transmission of a 5-µm-long VO_2_/SiN/BTO hybrid waveguide (Fig. 4) by means of 3D finite-difference time-domain (3D-FDTD) simulations (Supplementary note 1). On the one hand, in the insulating state and below 100 nm, the assumption of single-mode operation, along with the effective extinction coefficient provided by FEM simulations, is sufficient to predict the results of 3D-FDTD simulations; the insertion loss increases with the VO_2_ thickness as expected (Fig. 4a). However, for thicker VO_2_ layers, the insertion loss of the device reduces conversely to the trend predicted by propagation losses. This effect is caused by the excitation and significant coupling of higher optical modes supported by the hybrid waveguide. These higher-order modes feature much lower propagation losses, yielding an effective propagation loss value significantly lower than predicted by considering the excitation and coupling of the fundamental mode. On the other hand, in the metallic state, we observed a deviation between FEM and 3D-FDTD loss values as a function of the VO_2_ thickness and for gaps smaller than 150 nm (Fig. 4b). Such a variation is due to coupling losses, which are reduced to negligible values for gaps larger than 200 nm. Therefore, we choose a 100-nm-thick VO_2_ layer with a 200 nm gap as the optimal geometry for the hybrid waveguide to build our ELU activation function device. The optimal parameters are summarized in Table 1.

**Table 1:** Summary of optimized geometrical and optical parameters of the VO_2_/SiN/BTO hybrid waveguide.

VO_2_ thickness (nm)	Gap (nm)	Length (µm)	Propagation loss i-VO_2_ (dB/µm)	Propagation loss m-VO_2_ (dB/µm)
100	200	5	3.91	0.52

### Low-threshold power and bias-tunable photothermal response

In order to determine the nonlinear optical response of our device, we carried out steady-state thermo-optical simulations to obtain the temperature distribution of the VO_2_ patch as a function of the input power in the hybrid waveguide (Supplementary note 2), and thus the resulting output power.

At room temperature (20 °C), we achieved an ELU-like response with an optical power below 0.5 mW to initiate the IMT of VO_2_. As the input power is increased, the typical exponential response for this kind of activation function response is caused by the IMT of VO_2_ combined with the gradual variation of the temperature along the propagation direction. On the other hand, a linear input-output relationship is obtained for optical powers higher than 2.5 mW because the VO_2_ patch becomes fully metallic.

**Fig. 5:**
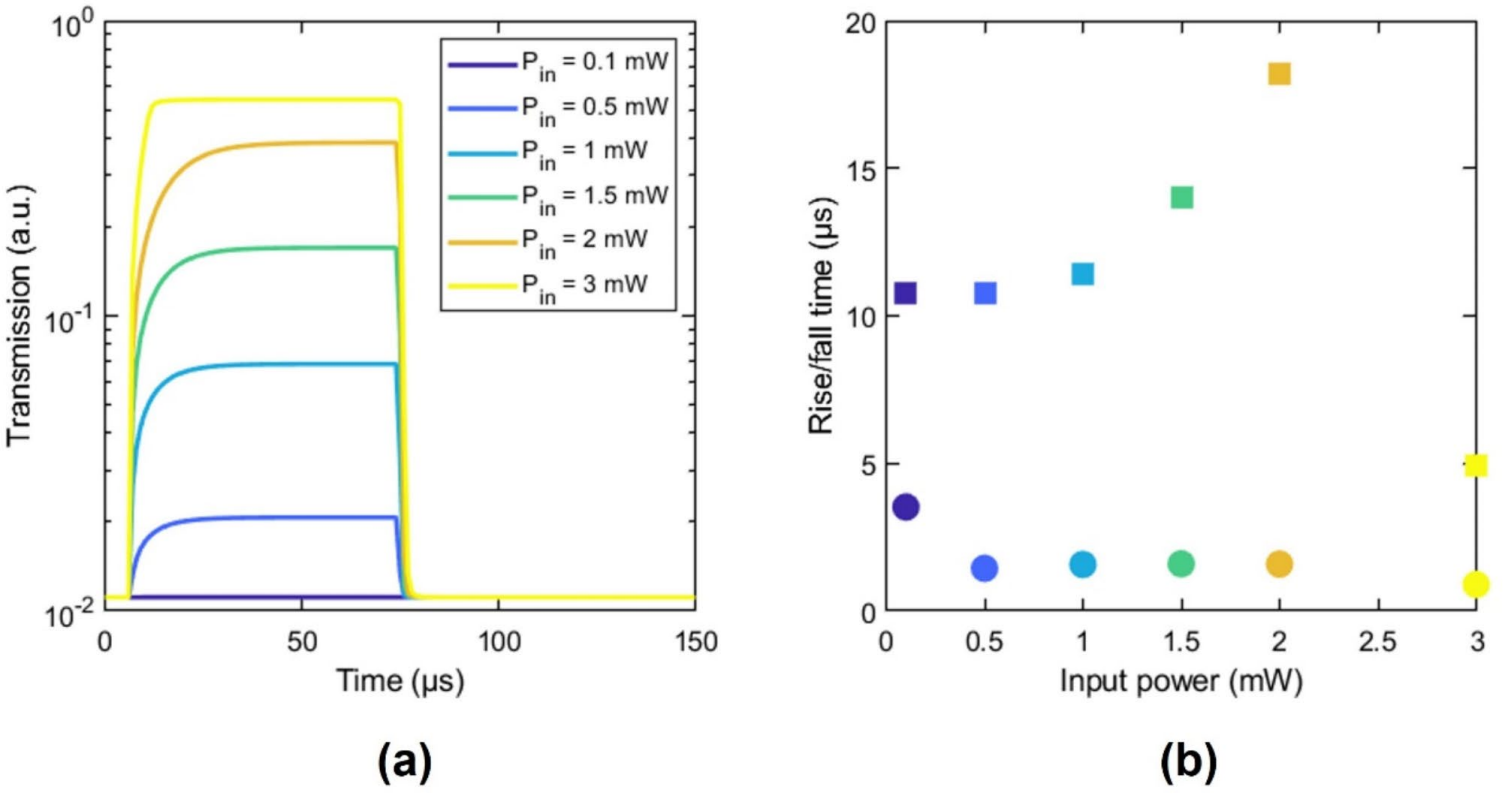
(**a**) Temporal response of the activation function device when a 50-µs-wide square optical signal with different peak power, P_in_, is launched into the VO_2_/SiN/BTO waveguide. (**b**) Associated rise (squares) and fall (dots) times of the activation function as a function of input power. Results are shown for a device background temperature of 20 °C (room temperature).

**Fig. 6:**
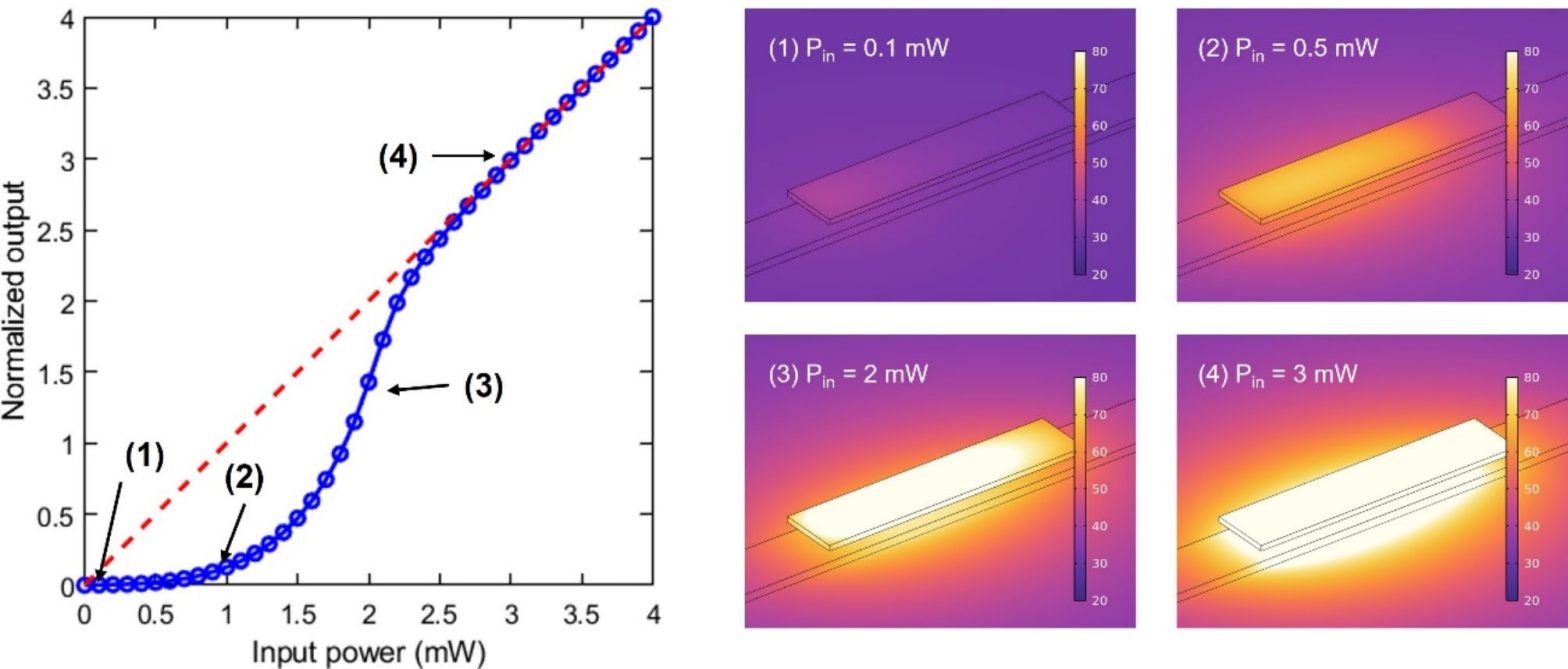
Normalized input-output power relationship and temperature distribution (in degC) of the hybrid waveguide in the different working regimes. Results are shown for a device background temperature of 20 °C (room temperature).

We investigated the temporal dynamics of our ELU-like device by carrying out time-domain thermo-optical simulations at room temperature (Supplementary note 2). To this end, we modeled the input power as a rectangular pulse of 50 µs to reach the steady state of the device. We determined the rise and fall time by applying the 10–90% rule in the transmission response of the device (Fig. 5a). For optical powers below the IMT threshold, the hybrid waveguide acts as a passive device since there is no variation of the VO_2_ refractive index, thus, although there is a heating and cooling duration, the temporal response of the activation function is instantaneous. In its nonlinear regime, the rise time there is an interplay between the IMT and the gradual heating of the VO_2_ patch. We observed a nonlinear relationship between the rise time and the peak power of the optical pulse (Fig. 5b). For optical powers below 1 mW, where the output power remains almost constant with the input power (Fig. 6), the rise time remains around 10 µs. For higher optical power, the rise time suffers a nonlinear dependence on the input power. Finally, above 2.5 mW, the rise time is reduced down to 5 µs since the temperature increase is sufficient to uniformly change the VO_2_ patch to the metallic state. In this case, the temporal response is mainly determined by the dimensions of the device and its thermal constants.

**Fig. 7:**
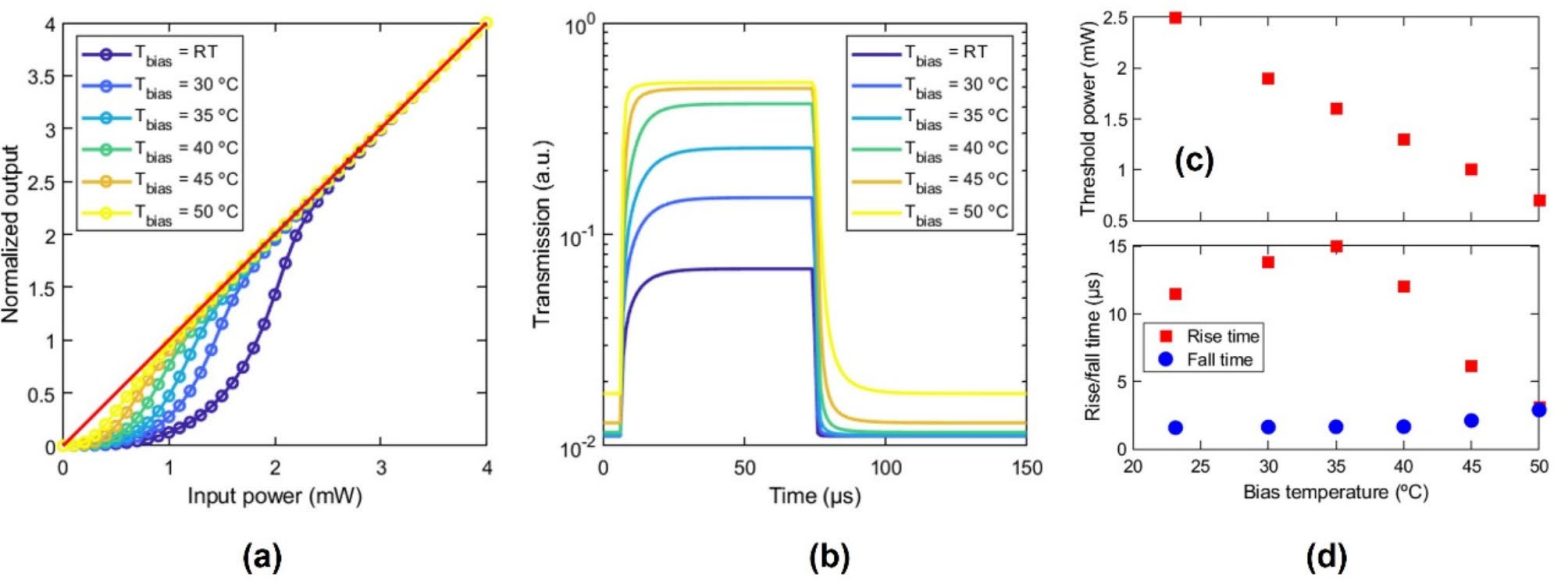
Performance of the proposed device for different bias temperatures, *T*_*bias*_. (**a**) Input-output relationship. (**b**) Temporal response under a 50-µs-wide optical pulse of 1 mW. (**c**) Threshold power and rise/fall times.

Since the working operation of the device is thermal based, the optical power for which the device’s optical response changes from nonlinear to linear (threshold power, $\:{P}_{th}$) can be tuned by changing the background temperature of the device, i.e., the device response can be biased. In this regard, as the background or bias temperature is increased, the activation function is shifted toward lower input powers (Fig. 7a) because the necessary temperature increment to induce the IMT is reduced. As a consequence, the temporal response of the device is also modified (Fig. 7b). Therefore, the threshold power and speed can be tuned as a function of the bias temperature (Fig. 7c), where sub-milliwatt threshold values and a few microsecond speeds could be achieved by setting the bias temperature in the vicinity of the IMT (~ 45 °C).

### Potential application in neural network architectures

We evaluated the performance of the proposed hardware ELU-like activation functions in an ANN architecture. The simulated nonlinear shapes of the activation functions were employed to train a convolutional neural network (CNN) model on the CIFAR-10 dataset. In general, the ELU activation function is a well-suited choice to train CNN models. ELU offers smoothness across its entire domain, ensuring continuous gradients that facilitate more stable and efficient gradient propagation during training. Unlike ReLU, which can suffer from dead neurons (zero gradients) and saturation issues with negative inputs, ELU allows negative values, enhancing the model’s ability to capture features in images.

**Fig. 8:**
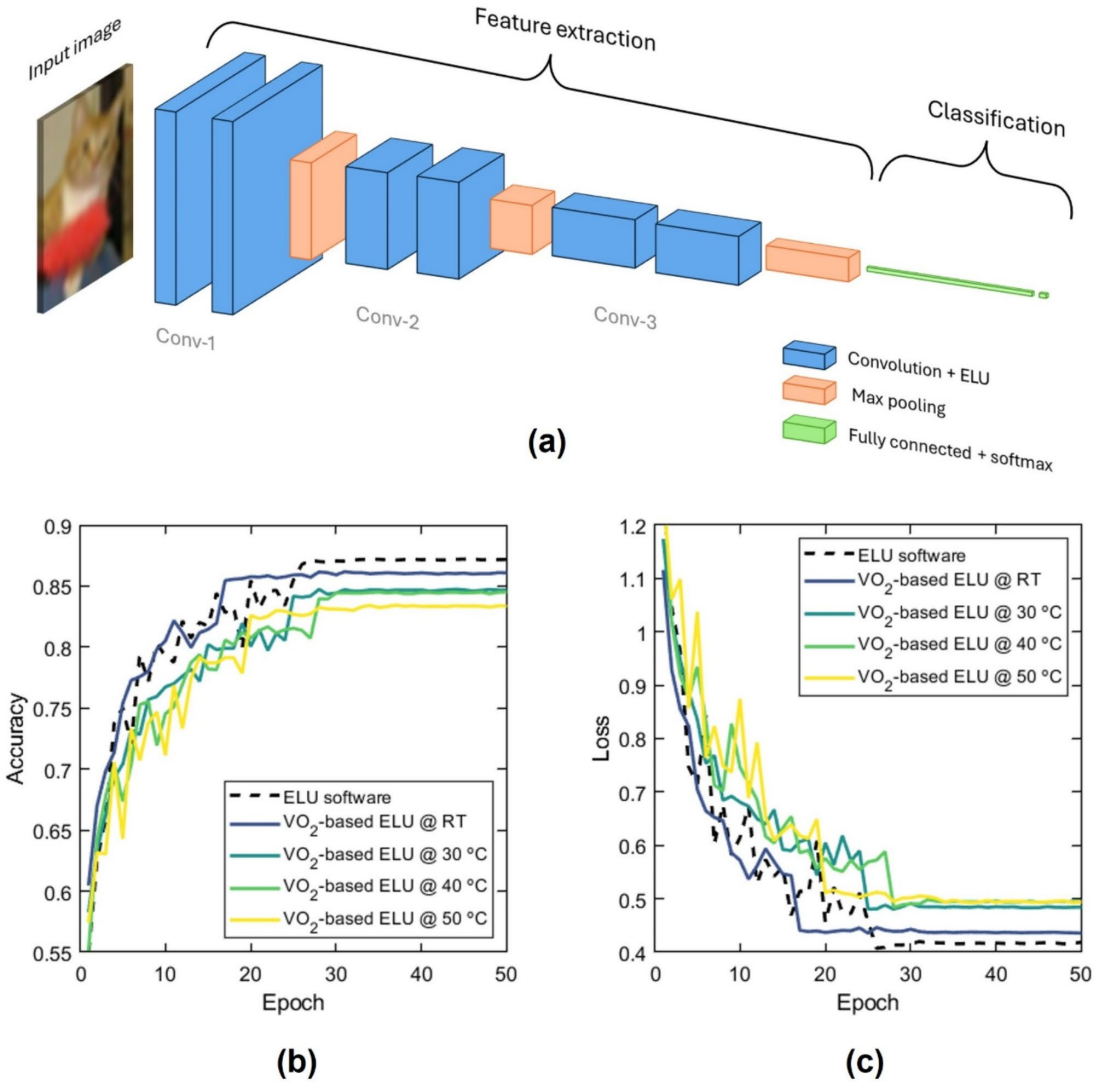
(**a**) Schematic of the CNN. (**b**, **c**) Validation (**b**) accuracies and (**c**) losses across epochs for various activation functions, including a custom ELU function simulating the hardware response of the device at different temperatures and software ELU.

The considered CNN model consists of three sets of convolutional layers, each followed by batch normalization and an activation function (Fig. 8a). The activation is varied to test different temperatures and compared with software ELU function implementation. These convolutional layers progressively increase in depth (32, 64, 128), allowing the model to extract hierarchical features from input images while mitigating issues like overfitting through the application of dropout layers after max-pooling operations. Max-pooling layers reduce spatial dimensions, aiding in feature extraction. Detailed information on implementation and parameters used for the layers of the CNN can be found in Supplementary note 3.

Figure 8b, c show the validation accuracy and model loss after 50 epochs of training the CNN. All functions result in high accuracy in the range of 80–85% (Fig. 8b), demonstrating that the proposed VO_2_/Si waveguide activation function can behave similarly to an ELU function. A slight degradation of accuracy and an increase in losses are observed when the device approaches the IMT temperature (Fig. 8c). This phenomenon can be attributed to the diminishing resemblance of the hardware response to the software ELU function (see Fig. 7a) and, thus, the loss of its inherent non-linear shape. Nonetheless, this small penalty comes with a great advantage in the threshold power and speed, as shown in Fig. 7d.

On the other hand, bias sensitivity and calibration can pose a challenge in photonic neural networks. In the context of using our VO_2_ activation function device, both hardware and software adaptive methods could be applied to improve the resilience of the neural network to external disturbances. On the hardware side, auto-calibration techniques using photonic feedback loops and thermal stabilization mechanisms can mitigate fabrication variations and maintain stable IMT behavior^50–52^. Complementary, hardware-aware training approaches on the software layer could incorporate the nonlinear response of the device into the network optimization, thereby enhancing the robustness and efficiency under varying operational conditions^53,54^.

## Discussion

The main benefit of VO_2_ is a power-efficient and strong photoinduced nonlinear response due to its IMT accompanied. This starkly contrasts with silicon or silicon nitride, which requires high powers or narrowband cavities to achieve comparable nonlinear effects. On the other hand, our proposed VO_2_ activation function device can be adapted to varying input power levels by thermally tuning the bias. This ensures efficient operation across different scenarios. The flexibility to adjust the transition point would also enable the device to maintain consistent performance, even under dynamic conditions. On the other hand, the tunability of the device allows it to operate at a low power threshold, which is essential for energy-efficient systems. This is particularly important in photonic neural networks, where minimizing power consumption with minimal penalty on the performance is a key design goal. Nonetheless, it must be noted that the photothermal triggering of the IMT in VO_2_ requires certain optical absorption to raise the temperature.

Compared to previous integrated photonic activation function devices (Table 2), our proposed device would benefit from a broad spectral operation due to the non-resonant approach and the utilization of VO_2_, which shows low wavelength dependence in their refractive index at telecom wavelengths^40,42^, while maintaining a compact footprint and low-threshold power such as all-optical activation function devices based on resonant and optical bandwidth-limited structures^29–31,35^. Temporal dynamics show a rise time as low as 5 µs, corresponding to a bandwidth of approximately 70 MHz. While this may not qualify as high-speed for certain ultra-fast computational tasks, it remains suitable for a wide range of moderate-speed operations. On the other hand, electro-optical activation function can benefit from using high-sensitivity photodiodes to detect low-power signals without necessitating resonant nonlinear structures^27,34–36^. However, the footprint is drastically increased considering the photodiode and associated electronic circuitry.

Despite the benefits of using VO_2_ for nonlinear optical devices, challenges associated with VO_2_ fabrication must be addressed for practical implementation. Mainly, achieving high-quality, reproducible, and high throughput of VO_2_ films is currently a challenge that must be addressed on the fabrication side. The synthesis of high-quality VO_2_ thin films for CMOS-compatible platforms is particularly challenging due to the material’s sensitivity to stoichiometric deviations and substrate conditions. Sputtering^55,56^, molecular beam epitaxy (MBE)^57,58^, and atomic layer deposition (ALD)^59,60^ are promising techniques for achieving uniform, high-quality films. These methods offer precise control over the oxygen-to-vanadium ratio, which is critical to ensuring the sharpness and reproducibility of the IMT. However, achieving the required phase purity and avoiding the formation of undesirable Magnéli phases remains a significant challenge, as the multiple oxidation states of vanadium (V^2+^, V^3+^, V^4+^, and V^5+^) make the process highly sensitive to growth conditions. Additionally, the grain size can significantly influence the crystallinity and IMT behavior of VO_2_ films^61^.

In conclusion, in this work, we have proposed an all-optical device for nonlinear activation functionalities in WDM photonic neural networks. Our device is based on the IMT of VO_2_ integrated into a SiN/BTO waveguide and exhibits a sub-milliwatt threshold, a compact footprint of 5 μm², and an EKU-like nonlinear shape. On the other hand, temperature tuning enables further optimization of speed and power efficiency. Moreover, performance evaluations using the CIFAR-10 dataset confirmed the device’s potential in CNN architecture. Therefore, our proposed device is particularly suitable for dense integrated photonics, potentially leading to significant improvements in the scalability and efficiency of all-optical neural networks.

**Table 2:** Survey of waveguide-based nonlinear activation function devices in integrated photonics.

References	Simulation/experimental	Technology	Implemented activation functions	All-optical/O-E-O	Optical bandwidth	Threshold power	Switching speed	Footprint
^27^	Exp.	ITO/Si EAM	N/A	O-E-O	Broad	N/A	N/A	5 μm
^28^	Exp.	Si MZI/MRR EOM	ReLU, Sigmoid	O-E-O	Limited	0.2 mW	N/A	N/A
^29^	Exp.	Si MZI-MRR	Sigmoid, Clipped ReLu, Radial-basis, Softplus	All-optical	Limited	N/A	N/A	N/A
^30^	Exp.	Ge/Si MRR	ReLU, ELU	All-optical	Limited	0.74 mW	100 kHz	N/A
^31^	Exp.	Si MRR	Softplus, radial basis, clipped ReLU, sigmoid	All-optical	Limited	0.08 mW	N/A	102 µm^2^
^32^	Exp.	Ge/Si DC	Not determined	All-optical	Broad	5.1 mW	70 MHz	N/A
^33^	Exp.	Graphene/Si MRR	Not determined	O-E-O	Limited	0.5 mW	N/A	N/A
^34^	Exp.	ITO/Graphene/Si EAM	Leaky ReLU	O-E-O	Broad	N/A	N/A	1.4 μm
^35^	Exp.	Si MRR	Sigmoid, radial basis, negative ReLU, softplus…	O-E-O and All-optical	Limited	< 0.1 mW	GHz	N/A
^36^	Sim.	TCO/Si EAM	Complex	O-E-O	Broad	N/A	100 GHz	N/A
This work	Sim.	VO_2_ on SiN/BTO waveguide	Tunable ELU	All-optical	Broad	0.5 mW	5 µs	5 µm^2^

Nonlinearity occurs in the waveguide.

*ITO* indium tin oxide, *Si* silicon, *EAM* electro-absorption modulator, *MZI* Mach-Zehnder interferometer, *MRR* microring resonator, *Ge* germanium, *DC* directional coupler, *TCO* transparent conductive oxide, *O-E-O* optical-electrical-optical, *N/A* not available.

## Electronic supplementary material

Below is the link to the electronic supplementary material.

Supplementary Material 1

## Data Availability

The datasets generated during and analysed during the current study are available from the corresponding author on reasonable request.
