# Supplementary material for: Sub-milliwatt threshold power and tunable-bias all-optical nonlinear activation function using vanadium dioxide for wavelength-division multiplexing photonic neural networks
Source: Sci Rep. 2025 Feb 15;15:5608. doi: 10.1038/s41598-025-90350-3 (PMC11829994; doi:10.1038/s41598-025-90350-3)
Supplement: Supplementary file 1 — Supplementary Material 1 [file 41598_2025_90350_MOESM1_ESM.pdf]

Sub-milliwatt threshold power and tunable-bias all-optical nonlinear activation function using vanadium dioxide for wavelength-division multiplexing photonic neural networks

Jorge Parra[1], Juan Navarro-Arenas[1,2], and Pablo Sanchis[1,]*

[1]Nanophotonics Technology Center, Universitat Politècnica de València, Camino de Vera s/n, 46022, Valencia, Spain

[2]Institute of Materials Science (ICMUV), Universitat de València, Carrer del Catedràtic José Beltrán Martinez 2, 46980, Valencia, Spain

[*]Corresponding author: pabsanki@ntc.upv.es

Supplementary information

Supplementary note 1. Optical simulations.

Table S1 shows the refractive indices considered for optical simulations at 1550 nm wavelength.

Table S1. Refractive indices of the materials at 1550 nm.

Air	BTO	SiN	SiO_2	i-VO_2 (T = 25 °C)	m-VO_2 (T = 80 °C)
1	2.285	2.015	1.45	2.765+j0.432	1.789+j2.574

We considered the values of the VO_2 refractive index as a function of the temperature reported in Ref.[40] . From those values, we calculated the effective refractive index for the reported temperatures (**Fig. S1**). Then, we fitted the effective refractive indices using the Maxwell-Garnett model [**Eq. (S1)**], where the volume fraction f is given by the Boltzmann function [**Eq. (S2)**].

$$\varepsilon_{EMT} = \varepsilon_i \frac{\varepsilon_m(1+2f) + \varepsilon_i(2-2f)}{\varepsilon_m(1-f) + \varepsilon_i(2+f)} \tag{S1}$$

$$f(T) = 1 - \frac{1}{1 + \exp\left(\frac{T - T_0}{\Delta T}\right)} \tag{S2}$$

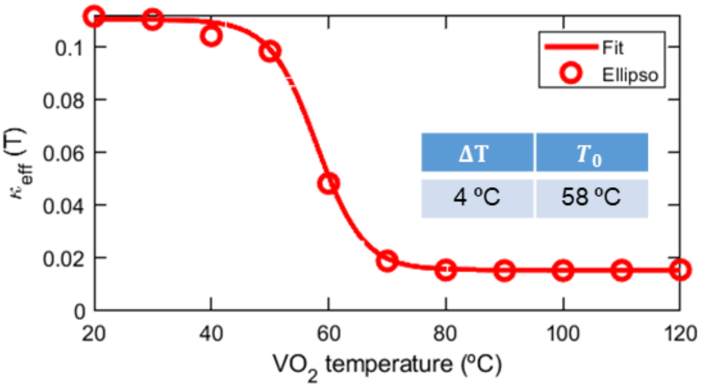

Fig. S1. Effective refractive index, κ_{eff}, of a SiN/BTO waveguide with a 100-nm-thick VO_2 on top spaced by a gap of 200 nm. The table shows the fitting parameters.

We employed a finite element method (FEM) eigenmode solver (FemSIM from RSoft) to calculate the optical modes. We used a nonuniform mesh consisting of a 30 nm × 30 nm bulk grid with minimum divisions of 10 points in both the x and y axes. We used a symmetric boundary condition at x = 0 to alleviate time and computational cost. The simulated domain comprised from 0 µm to +1 µm in the x-axis, whereas from -1 µm to +1 µm in the y-axis.

The propagation loss, α, was calculated from the complex effective refractive index as:

$$\alpha = \frac{20\log_{10}(e)\, 2\pi\kappa_{\text{eff}}}{\lambda} \qquad (S3)$$

where κ_{eff} is the imaginary part of the complex effective refractive index, and λ is the working wavelength used in the simulation (1550 nm).

3D finite-difference time-domain (3D-FDTD) simulations using the FullWAVE tool from RSoft were conducted to calculate the insertion loss (coupling loss + propagation loss) of the device. In the XY plane, we used the same simulation configuration as for the calculation of the optical modes. In the propagation direction (z-axis), we used a 30 nm bulk grid. A perfectly matched layer (PML) was employed as a boundary condition in the remaining boundaries. Each PML boundary consisted of 10 PML cells to avoid reflections. Overlap monitors for the fundamental mode of the SiN/BTO waveguide were placed before and after the device to determine the insertion loss.

Supplementary note 2. Thermal simulations.

Table S2 shows the thermal constants considered for thermal simulations. Thermo-optical contributions from SiN, BTO, and SiO_2 were considered to be negligible.

Table S2. Thermal constants of the materials.

	Si	VO_2	SiO_2	SiN	BTO
Thermal conductivity (W/(m K))	148	3.5	1.38	18.5	2.61
Density (kg/m^3)	2330	4571	2203	3100	5840
Heat capacity (J/(kg K))	703	656	709	788	434

3D thermal simulations were conducted by solving the heat conduction equation in the steady-state and the time domain with the Heat Transfer Module of COMSOL Multiphysics simulation tool. We modeled the VO_2 patch as a heat source, where heating arises from the absorption of the optical evanescent field. We approximated the heat source, Q, spatial distribution in the XY plane to be uniform, while in the z-axis (propagation direction), we considered the exponential decay caused by the evanescent field, resulting thereby in the following expression:

$$Q(T,z)=\frac{P\Gamma}{wt}\frac{4\pi\kappa_{\text{eff}}(T)}{\lambda}\exp\left(-\frac{4\pi\kappa_{\text{eff}}(T)}{\lambda}z\right). \qquad (S4)$$

where P is the input power of the waveguide, Γ is the coupling factor between the SiN/BTO and VO_2/SiN/BTO waveguides, w and t are the width and thickness of the VO_2 patch, respectively, λ is the working wavelength (1550 nm), $\kappa_{\text{eff}}(T)$ is the value of the effective absorption coefficient of the optical mode propagating along the hybrid waveguide as a function of the temperature [**Fig. (S1)**], and z is the position alongside the VO_2 patch from the beginning (z=0) to the end (z=5 μm) of the hybrid waveguide. As a consequence, the VO_2 patch suffers a nonlinear and nonuniform change in its refractive index profile due to the in-plane approach and the insulator-metal transition of VO_2. Thus, upon reaching the steady state of the device, the optical loss can be determined by FDTD.

The heat conduction equation becomes highly nonlinear due to the presence of VO_2 as a heat source; thereby, a two-step study was defined for the stationary case. First, the temperature distribution was calculated by considering the VO_2 in the insulating state. Then, those results were set as initial values for solving the thermal response of the device considering its thermo-optical expression [**Eq. (S4)**]. On the other hand, for the time-dependent case, we solved the temperature distribution using **Eq. (S4)** and a time step 1/50 smaller than the width of the applied excitation pulse.

Regarding the simulation domain and settings for conducting thermal simulations, the length of the SiN/BTO waveguide extended to 20 μm (**Fig. S2**). The influence of the silicon substrate was taken into account, setting a size (width × height) of 20 μm × 20 μm. The

height of the SiO_2 under- and upper-cladding was 3 µm and 1 µm, respectively. A non-uniform tetrahedral mesh was employed. The VO_2 patch was discretized with element sizes between 100 and 50 nm with a minimum division of 5 points in its thickness (y-axis). The SiN domain was defined by elements ranging between 100 and 200 nm. Finally, the remaining domains consisted of elements between 100 nm and 3 µm. Convective heat flux was set as the boundary condition on top of the upper cladding with a heat transfer coefficient $h = 5 \text{ W m}^{-2}\text{K}^{-1}$. Temperature boundary condition was applied to the remaining boundaries with a value of 293.15 K (20 °C).

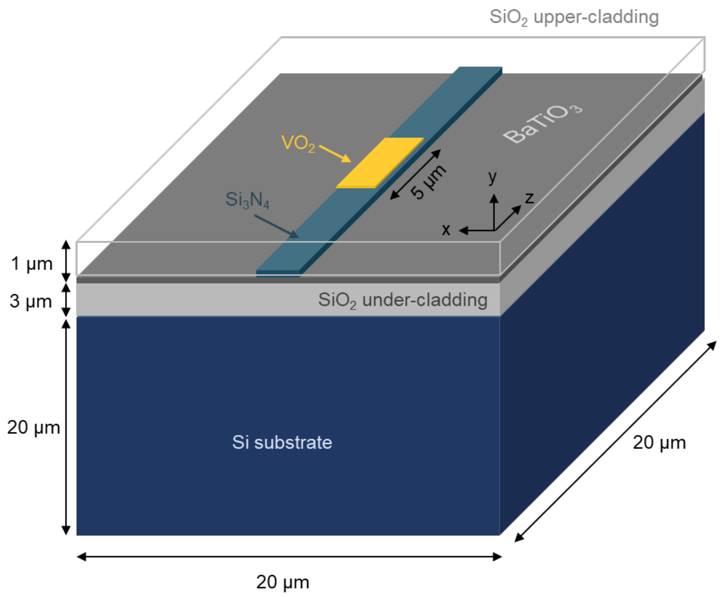

Fig. S2. Illustration (not to scale) of the 3D simulated region to conduct thermal simulations.

Supplementary note 3. Convolutional neural network simulations.

The exponential linear unit (ELU) function is defined as:

$$f(x) = \begin{cases} x, & x \geq 0 \\ a(e^x - 1), & x < 0 \end{cases} \qquad (S5)$$

In our simulations, we have established a reference point at $x = 0$, where the ELU transitions from its nonlinear regime ($x < 0$) to a linear response ($x \geq 0$). To achieve this behavior, we implemented a threshold-based approach in the convolutional neural network (CNN) simulations. Optical power to the device was normalized and shifted such that $x = 0$ corresponded to the transition point between the linear and nonlinear responses. This allowed us to simulate the ELU-like activation function while respecting the physical constraints of the thermo-optic mechanism. In practical implementation on an integrated photonic platform, the shift required to emulate the ELU activation function ($x = 0$ as the

transition point) could be achieved using a thermal or optical bias, either to set the desired operating point or effectively shifting the input power range, respectively.

Table S3. Description of the layers comprising the CNN.

Layer Name	Height	Width	Depth	Filter Height	Filter Width
Input Layer	32	32	3	-	-
Conv-1.1	32	32	32	3	3
Conv-1.2	32	32	32	3	3
MaxPooling-1	16	16	32	2	2
Conv-2.1	16	16	64	3	3
Conv-2.2	16	16	64	3	3
MaxPooling-2	8	8	64	2	2
Conv-3.1	8	8	128	3	3
Conv-3.2	8	8	128	3	3
MaxPooling-3	4	4	128	2	2
Fully Connected (Dense)	1	1	512	-	-
Output Layer (Softmax)	1	1	10	-	-
